# Healthcare resource utilization and costs for multiple sclerosis management in the Campania region of Italy: Comparison between centre-based and local service healthcare delivery

**DOI:** 10.1371/journal.pone.0222012

**Published:** 2019-09-19

**Authors:** Marcello Moccia, Andrea Tajani, Rosa Acampora, Elisabetta Signoriello, Guido Corbisiero, Adriano Vercellone, Primo Sergianni, Francesca Pennino, Roberta Lanzillo, Raffaele Palladino, Antonio Capacchione, Vincenzo Brescia Morra, Giacomo Lus, Maria Triassi

**Affiliations:** 1 Multiple Sclerosis Clinical Care and Research Centre, Department of Neuroscience, Reproductive Science and Odontostomatology, “Federico II” University, Naples, Italy; 2 Queen Square MS Centre, UCL Queen Square Institute of Neurology, University College London, London, United Kingdom; 3 Department of Public Health, “Federico II” University, Naples, Italy; 4 Primary Care and Local Service Unit, Local Healthcare Services “Napoli 3 Sud”, Naples, Italy; 5 Multiple Sclerosis Centre, II Neurology Clinic, "Luigi Vanvitelli" University of Campania, Naples, Italy; 6 Local Healthcare Service 57, Local Healthcare Services “Napoli 3 Sud”, Naples, Italy; 7 Department of Pharmaceutics, Local Healthcare Services “Napoli 3 Sud”, Naples, Italy; 8 Department of Primary Care and Public Health, Imperial College, London, United Kingdom; 9 Merck Serono S.p.A., an affiliate of Merck KGaA, Darmstadt, Germany, Via Casilina, Rome, Italy; Keck School of Medicine of the University of Southern California, UNITED STATES

## Abstract

**Background:**

Multiple sclerosis (MS) requires multidisciplinary management. We evaluated differences in healthcare resource utilization and costs between Federico II and Vanvitelli MS Centres of Naples (Italy), representative of centralised (i.e., MS Care Unit) and local service-based models of multidisciplinary care, respectively.

**Methods:**

We included MS patients continuously seen at the same local healthcare services and MS Centre (Federico II = 187; Vanvitelli = 90) from 2015 to 2017. Healthcare resources for MS treatment and management were collected and costs were calculated. Adherence was estimated as the rate of medication possession ratio (MPR) during 3-years of follow-up. Mixed-effect linear regression models were used to estimate differences in all outcomes between Federico II and Vanvitelli.

**Results:**

Patients at Federico II had more consultations within the MS centre (p<0.001), blood tests (p<0.001), and psychological/cognitive evaluations (p = 0.040). Patients at Vanvitelli had more consultations at local services (p<0.001). Adherence was not-significantly lower at Vanvitelli (p = 0.060), compared with Federico II. Costs for MS treatment and management were 10.6% lower at Vanvitelli (12417.08±8448.32EUR) (95%CI = -19.0/-2.7%;p = 0.007), compared with Federico II (15318.57±10919.59EUR).

**Discussion:**

Healthcare services were more complete (and expensive) at the Federico II centralised MS Care Unit, compared with the Vanvitelli local service-based organizational model. Future research should evaluate whether better integration between MS Centres and local services can lead to improved MS management and lower costs.

## Introduction

Multiple sclerosis (MS) is a chronic disease whose typical onset is in young adulthood, thus representing a source of physical, cognitive, social and economic burden through the lifetime [1–4]. The natural history of MS has changed with the introduction of disease-modifying treatments (DMTs), including a wide range of drugs with different mechanisms of action and efficacy/safety profiles [1,5]. As a consequence, MS treatment currently requires access to a large set of investigations, including magnetic resonance imaging (MRI), immunological tests, infusion therapy, and specialist assessments [6]. Not least, patients will also inevitably require specialized multidisciplinary symptomatic management (e.g., spasticity, bladder, bowel, pain, fatigue, cognitive impairment) [7].

MS treatment and management has benefited from the creation of MS Care Units, based on integrated patient-care (diagnosis, treatment and follow-up), with MS neurologists and nurses working with other specialists within formalized workup protocols and offering a high number of activities to MS patients in a “single stop, single shop” approach [8]. MS Care Units with a centralised multidisciplinary approach are continuously up-to-date with the evolving scenario of MS management, and, as such, can enhance the efficacy of therapy, provide better patient overall satisfaction, and, not least, be cost-effective for the society [9]. However, the latter is difficult to document in the short term and would require socio-economic studies with long-term observation [8].

Many epidemiological studies have classified Italy as a high-risk area for MS, with more than 100,000 MS patients, and, thus, MS represents an economic challenge for the Italian National Healthcare System, based on the universal coverage of healthcare needs [10]. DMTs are homogenously accessible at national level, whereas other services are below the expected standard of care (e.g., rehabilitation, psychological support) [4,11,12]. However, there are differences in the pathways for healthcare delivery, with variable involvement of MS Centres and local services [13], possibly affecting MS management and costs. A number of MS healthcare pathways have been suggested and applied to different hospitals and Regions, also with the contribution of the Italian MS Society and the Italian Society of Neurology, but their economic viability is still unknown.

In the present 3-year cohort study, we aim to evaluate (1) differences in healthcare resource utilization and costs between two MS Centres, each of which being representative of either centralised (i.e., MS Care Unit) or local service-based model of healthcare delivery; and (2) possible associations between costs and MS clinical features.

## Methods

### Study design and population

**Fig 1:**
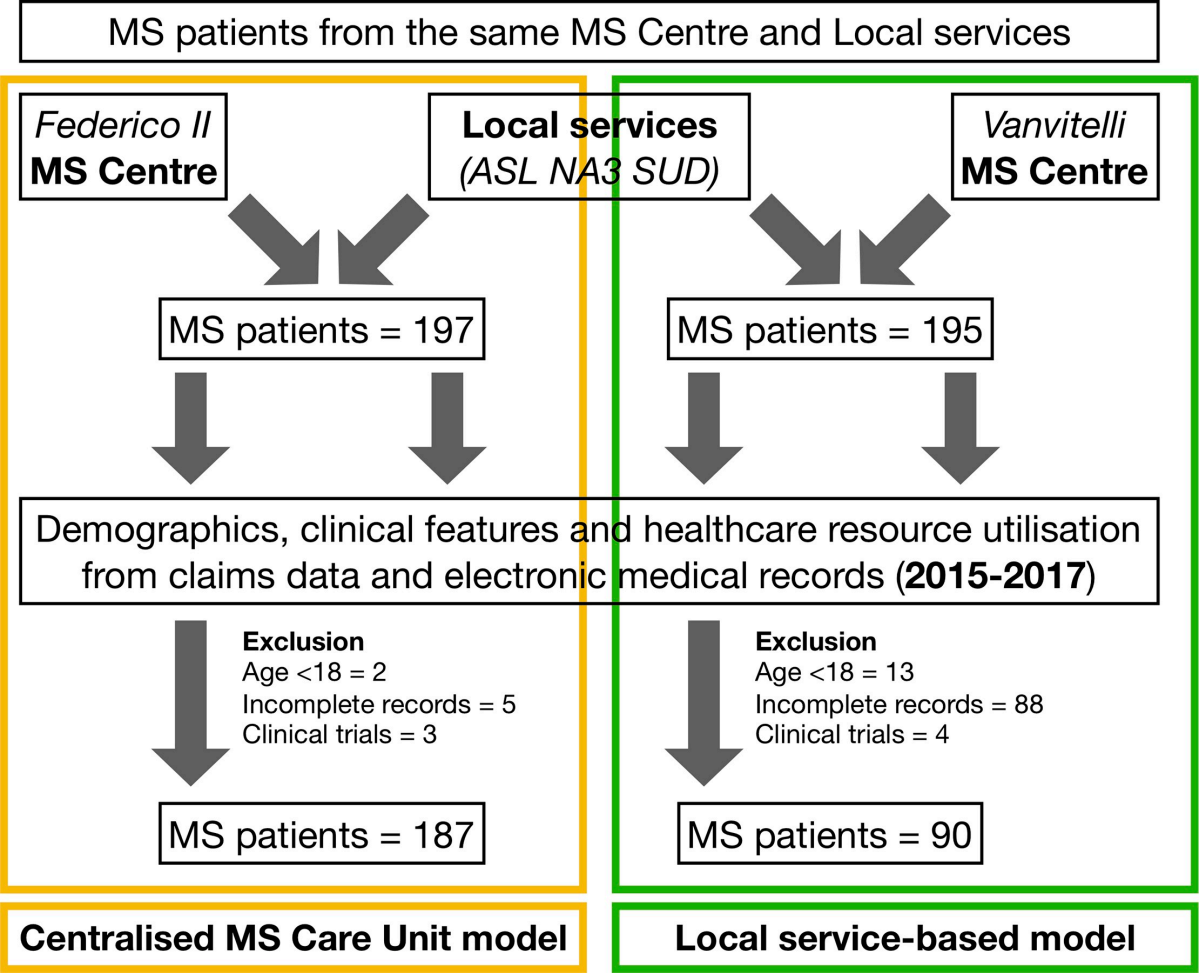
Cohort development and patient disposition. Figure shows cohort development, with inclusion of MS patients regularly followed-up at the same MS Centre (either Federico II or Vanvitelli University) and local healthcare services (3^rd^ local healthcare agency for the south of Naples (ASL NA3 SUD)). Demographics, clinical features, and healthcare resource utilisation were extracted from claims data and electronic medical records within the study period (2015–2017). Federico II MS Centre was highly centralised in healthcare delivery, with a MS Care Unit organizational model (yellow), whereas Vanvitelli was based on local services for anything other than neurological consultations and day-service infusion (green). Reasons for exclusion are reported.

The present observational cohort study is a retrospective analysis of prospectively collected data (claims data and electronic medical records). Inclusion criteria were: 1) diagnosis of MS [14]; 2) patients continuously seen at the same MS Centre (either Federico II or Vanvitelli University) and local services (3^rd^ local healthcare agency for the south of Naples (ASL NA3 SUD)) within the study period (2015–2017); 3) DMT prescription during the study period. Exclusion criteria were: 1) incomplete clinical records (e.g., absence of recorded access within either MS centre or local services during the study period); 2) age <18 years; 3) participation in clinical trials for DMTs during the study period (Fig 1).

ASL NA3 SUD was selected as local healthcare service provider, because it is largely representative of both urban and rural areas, and provides its patients with homogenous services on its reference area. As such, included MS patients from both Federico II and Vanvitelli MS Centres had full access to local ASL NA3 SUD healthcare services.

MS Centres based at the Federico II and the Vanvitelli University were selected due to their differences in the site of healthcare service delivery. The Federico II University MS Centre has a highly centralised pathway of healthcare delivery for the management of DMTs and complications, as suggested for the MS Care Unit [8]; healthcare services and consultations are delivered within the same hospital upon request of MS specialists. On the contrary, the Vanvitelli University MS Centre refers patients to local services, through the general practitioner (GP), for any service other than neurological consultations and day-service infusion (if needed).

In compliance with current Italian applicable laws and regulations, considering that all clinical assessments were part of clinical practice in a University setting and that the retrospective analysis included anonymized data, specific ethics approval was not required. Patient unique identifier code was fully anonymised by the regulatory agency at ASL NA3 SUD before releasing the datasets. All subjects signed the general informed consent form for current data protection regulation (GDPR EU2016/679), authorizing the use of anonymized data collected routinely as part of the clinical practice. The study was performed in accordance with the good clinical practice and the Declaration of Helsinki.

### Healthcare utilizations and costs

DMTs were prescribed in accordance with regulatory indications for clinical practice (Italian and European Medicines Agencies). MS Centres and local services were under the direct control of the Campania Region Healthcare Regulatory Agency and, as such, provided patients with similar services, being different only in the delivery (within the MS Centre or on the local services).

Healthcare services for the present study included DMTs, general hospital costs, staff involved in DMT administration (either for training the patient and his/her caregiver in self-administration, or for inpatient administration procedures), neurological visits, other specialist consultations related to DMT safety or MS clinical features (e.g., ophthalmology, rehabilitation), MRI procedures, laboratory exams, psychological/cognitive evaluations (either diagnostic or therapeutic), performed in accordance with current guidelines and clinical practice [2,3,15,16]. The analysis dataset was obtained by linking clinical databases from local services (ASL NA3 SUD) and University-based MS Centres in order to obtain full view of healthcare resource utilization.

Medication possession ratio (MPR) was calculated as an indirect measure of adherence (MPR = (medication supply obtained during follow-up period/medication supply expected during follow-up period)*100). Expected refill interval was calculated for each DMT. In the clinical practice, refill was electronically checked and early refills were not allowed, thus limiting the risk of overestimated adherence [17].

Healthcare costs were inflated to the most recent values (2017), in order to avoid variations in price per unit of service through different years, obtained from the National Drug Formulary for DMT costs (Italian Drug Agency), and from the National Tariffs for Healthcare of the Italian National Health System for resource utilization costs (Italian Ministry of Health), as previously performed in similar studies on Italian MS populations [2,3,18]. Healthcare costs were then annualized (annual healthcare costs = sum of healthcare costs from the whole study period/years of study). We based our estimates on national prices and not on local prices (that could imply a discount) to improve result generalizability.

### Demographic and clinical variables

Age and sex were recorded. Study duration was calculated from the first (2015) to the last (2017) recorded visit in either MS Centre or local service datasets. During the whole study period, MS patients attended the MS Centre and local services for visits which were scheduled according to the clinical practice for the follow-up of their disease and treatment, or for the occurrence of a clinical relapse, and were evaluated for:

- Clinical course: the cohort was classified into primary progressive (PP), relapsing remitting (RR), or secondary progressive (SP) MS [19];
- Occurrence of clinical relapse: relapsing patients presented with a range of motor/sensory symptoms and met commonly used standards for relapse as determined by clinical neurologists; the cohort was categorized according to the occurrence of a clinical relapse or not;
- Expanded Disability Status Scale (EDSS) progression: EDSS was evaluated at baseline and, then, EDSS progression was recorded (1-point progression for baseline EDSS≤5.5, or 0.5-point progression for baseline EDSS≥6.0); EDSS progression was confirmed after 6 months, and was independent from the occurrence of relapses; the cohort was categorized according to EDSS progression or not [19,20].

Neurological examination was performed by EDSS certified neurologists in both MS Centres. Criteria for defining clinical course, relapses and EDSS progression were preliminarily agreed between MS Centres, in accordance with current clinical definitions [19,20].

### Statistical analyses

For descriptive purposes, mean (and standard deviation), number (and percent), and median (and range) were calculated for different study variables, and differences between the Federico II and the Vanvitelli MS Centre, and between DMTs were preliminary explored using t-test and chi-square test, as appropriate.

For modelling purposes, comparisons between the Federico II and the Vanvitelli MS Centre in healthcare resource utilization, adherence and annualized costs (aim 1) were explored by using mixed-effect linear regression models, with a random subject intercept, and using age, sex, baseline EDSS, disease phenotype, study duration and DMT as covariates. Similarly, associations between MS clinical features and annualized costs (aim 2) were explored by using mixed-effect linear regression models, with a random subject intercept, and using age, sex, study duration, disease phenotype, DMT and MS Centre as covariates. Distribution of healthcare costs was preliminarily analysed. Considering that data was right-skewed, cost variables were log-transformed. Results were presented as “percentage difference” (PD) using the following formula Percentage difference = (e^Ln(Regression Coeff.)^ - 1)*100.

Statistical analyses were performed with Stata 15.0. Results were considered statistically significant for p<0.05.

### Availability of materials and data

Due to legal and ethics restrictions on sharing data containing potentially identifying patient information, as from the GDPR (EU 2016/679), data are available upon request to the Department of Public Health, Federico II University, Naples, Italy (triassi@unina.it).

## Results

We included 277 MS patients from the original cohort of MS patients within ASL NA3 SUD and regularly followed up at either Federico II (n = 187/197, 94.9%) or Vanvitelli MS Centres (n = 90/195, 46.1%). Cohort development and patient disposition are reported in Fig 1. Patients excluded due to incomplete clinical records presented with similar baseline demographic and clinical features, and DMT utilization, when compared with patients included in study analyses.

Patients at Vanvitelli MS Centre presented with lower EDSS and shorter follow-up, when compared with Federico II (Table 1). Federico II and Vanvitelli MS Centres presented with not statistically different DMT utilization during the study period (p = 0.076) (Table 2).

**Table 1:** Demographic and clinical characteristics.

		Federico II (n = 187)	Vanvitelli (n = 90)	p-values
**Age, years**		42.1±10.5	40.9±10.7	0.371
**Sex, female**		118 (63.1%)	58 (64.4%)	0.828
**Study duration, years**		2.8±0.4	2.7±0.5	0.036*
**Clinical phenotype**	**PPMS**	7 (3.7%)	7 (7.8%)	0.210
	**RRMS**	160 (85.6%)	70 (77.8%)	
	**SPMS**	20 (10.7%)	13 (14.4%)	
**Baseline EDSS**		3.5 (1.5–7.0)	1.5 (1.0–7.0)	<0.001*
**Follow-up EDSS**		3.5 (1.5–8.0)	1.5 (1.0–7.0)	<0.001*
**EDSS progression**		42 (22.5%)	18 (20.4%)	0.707
**Relapse occurrence**		51 (27.3%)	17 (18.9%)	0.129
**Number of relapses**		0.379±0.740	0.244±0.605	0.133

Table shows demographic and clinical features from Federico II and Vanvitelli MS Centres. P-values are reported from t-test and chi-square test, as appropriate (* indicates p<0.05).

**Table 2:** DMT utilization at Federico II and Vanvitelli MS centres.

	Federico II (n = 187)	Vanvitelli (n = 90)	DMT cost (EUR/month)
**Alemtuzumab**	7 (2.6%)	1 (0.8%)	9774.09
**Dimethyl Fumarate**	20 (7.2%)	14 (11.2%)	851.76
**Fingolimod**	42 (15.0%)	20 (16.0%)	1396.92
**Glatiramer acetate 40mcg**	17 (6.1%)	16 (12.8%)	657.72
**Interferon-β1a 30mcg im**	41 (14.8%)	13 (10.4%)	690.00
**Interferon-β1a 22mcg sc**	8 (2.9%)	4 (3.2%)	689.80
**Interferon-β1a 44mcg sc**	45 (16.1%)	16 (12.8%)	919.44
**Interferon-β1b (Betaferon)**	35 (12.5%)	14 (11.2%)	690.00
**Interferon-β1b (Extavia)**	4 (1.4%)	5 (4.0%)	630.00
**Natalizumab**	42 (15.0%)	9 (7.2%)	1551.40
**Peg-Interferon-β1a**	4 (1.4%)	3 (2.4%)	755.48
**Teriflunomide**	14 (5.0%)	10 (8.0%)	732.20

Table shows DMT utilization during the study period at Federico II and Vanvitelli MS Centres (including DMT switch). Cost for each DMT (per month) is reported (descriptive results).

### Healthcare resource utilization and costs

Patients at Federico II MS Centre had more consultations within the MS centre, blood tests and psychological/cognitive evaluations (either diagnostic or therapeutic). Patients at Vanvitelli MS Centre had more consultations at local services. Adherence (MPR) was not-significantly lower at Vanvitelli MS Centre (-1.0%), when compared with Federico II (Table 3). Annualized costs for MS treatment and management were 10.6% lower at Vanvitelli MS Centre (absolute annualized cost per patient = 12417.08±8448.32 EUR–around 14000 USD) (adjusted PD = -10.6%; 95%CI = -19.0/-2.7%; p = 0.007), when compared with Federico II (absolute annualized cost per patient = 15318.57±10919.59 EUR–around 17000 USD), independently from study covariates (age, sex, baseline EDSS, disease phenotype, study duration and DMT).

**Table 3:** Healthcare resource utilization and adherence.

	Federico II	Vanvitelli	Coeff	95%CI		p-values
				Lower	Upper	
**Consultations within the MS Centre**	8.2±2.6	3.0±1.7	-5.732	-6.496	-4.969	<0.001*
**Consultations within local services**	0.6±1.1	7.0±2.9	6.731	6.048	7.413	<0.001*
**Blood tests**	10.2±2.6	3.4±1.1	-5.169	-5.980	-4.357	<0.001*
**MRI exams**	1.5±1.6	0.8±0.6	-0.492	-1.034	0.048	0.075
**Psychological/cognitive evaluation**	3.6±0.8	3.0±1.9	-0.264	-0.516	-0.012	0.040*
**Adherence (MPR)**	99.2±0.1%	98.0±0.1%	-1.0%	-2.2%	0.1%	0.060

Table shows descriptive healthcare resource utilization and adherence during the study period at Federico II and Vanvitelli MS Centres. Adjusted coefficients (Coeff), 95%CI and p-values are presented from mixed-effect linear regression models, with a random subject intercept, and including age, sex, baseline EDSS, disease phenotype, study duration and DMT as covariates (* indicates p<0.05).

### Costs and clinical features

Across MS centres, annualized total costs were 20.7% higher in RRMS, and 18.2% higher in SPMS, when compared with PPMS. Annualized costs were 5.4% higher in patients presenting with a relapse during the study period, when compared with patients without relapses, and 5.1% higher in patients with EDSS progression, when compared with patients without EDSS progression (Table 4). Each point higher baseline EDSS was associated with 1.6% higher annualized costs (adjusted PD = 1.6%; 95%CI = 0.4/2.9%; p = 0.006). Aforementioned results were independent from study covariates (age, sex, study duration, disease phenotype, DMT and MS Centre).

**Table 4:** Costs and clinical features.

		Cost	PD	95%CI		p-values
		(EUR/year)		Lower	Upper	
**Disease subtype**	PPMS	9057.58±4337.64	reference			
	RRMS	14.866.75±10326.44	20.7%	8.9%	33.8%	<0.001*
	SPMS	13057.95±11099.66	18.2%	6.6%	31.1%	0.001*
**EDSS progression**	No	14109.94±9901.89	reference			
	Yes	15641.65±11795.55	5.1%	0.1%	10.5%	0.047*
**Relapse occurrence**	No	12937.48±8283.76	reference			
	Yes	18088.73±13435.92	5.4%	1.1%	9.9%	0.012*

Table shows descriptive absolute annualized cost per patient in relation to different disease outcomes (10000 EUR correspond to around 11000 USD). Adjusted percent difference (PD), 95%CI and p-values are presented from mixed-effect linear regression models, with a random subject intercept, and including age, sex, study duration, disease phenotype, DMT and MS Centre as covariates (* indicates p<0.05).

## Discussion

We performed a comprehensive evaluation of MS-related healthcare resource utilization and costs in a selected geographical and healthcare area (ASL NA3 SUD, Naples, Italy). Overall, healthcare resource utilization and costs were lower when most of the healthcare delivery occurred within local services, when compared with centralised delivery in a MS Care Unit organizational model. Healthcare resource utilization and costs were mainly driven by RRMS patients and by more severe disease features (e.g., relapses, disability), requiring higher number of assessments and more expensive DMTs.

Higher healthcare resource utilization and costs occurred within the centralised model of healthcare delivery, due to higher number of consultations and exams, repeated in both MS centre and local services, when compared with local service-based model. This result was independent from a number of covariates, including disease severity and DMT utilization, that were similar in the two healthcare models and were also accounted for in the statistical analyses. Considering the effort in developing a centralised model of healthcare for MS, this result could be read as rather disappointing, but was expected by Sorensen and colleagues on their preliminary description of the ideal MS Care Unit [8]. Rather than repetitions in actual services, we observed more complete healthcare within the centralized model (e.g., number of MRIs repeated one year apart, consultations for DMT safety or for concomitant issues). Also, specialty clinics are staffed differently than local clinics and, as such, higher costs are expected. Not least, in our study, after pragmatic evaluation of quality related aspects of healthcare resource utilization, authors agreed that poor coordination between MS Centre and local services could have been responsible for lower efficiency with higher healthcare resource utilization and costs in the centralised model of care. In an appropriate healthcare setting, case managers are responsible for coordination and integration of care between central and local services, ultimately leading to uniformity of healthcare services and controlled prescriptions of services [21,22]. At the Federico II MS Centre, we are currently developing a digital case manager possibly allowing better integration of services and reduced costs [9]. In the meantime, the model based on local services remains more financially viable.

In the present cohort, relapse occurrence and EDSS progression were associated with higher costs. Relapses are a well-known cause of higher healthcare resource utilization and costs, not only because of the direct consequences of a relapse, but also for hospital admissions and change of DMTs [4,23–26]. Disability at diagnosis and its progression over time can be considered a marker of a more aggressive disease, deserving more active and expensive treatments [27], and have already been described as one of the main determinants of the economic burden of MS [2]. In advanced stages of MS, costs are mainly driven by hospital admissions, palliative treatments and informal care [4,28]. As such, diagnosis in early phases of the disease and appropriate treatment could be effective in modifying the natural course of MS and, so, in avoiding its costly consequences [29]. Not least, based on our findings, policy makers could better plan resource allocations for managing MS, especially in its advanced stages. Our real-life cohort also included progressive MS patients with active disease [19], although costs were mainly driven by RRMS.

Of note, we found a trend towards a higher adherence rate (MPR) in the centralised model of care, when compared with local services. The lack of significance could be due to the relatively high adherence rates, as frequently described in Italian MS populations [30], inevitably requiring higher sample size to detect significant differences [31]. However, we hypothesize that a centralised model of care could positively impact on MS patients, as reflected by higher adherence rates.

Limitations of this study include the lack of patient-reported outcome and experience measures, that should be considered in the future to estimate patient satisfaction towards different healthcare delivery models [32]. We only included patients on DMTs and, thus, healthcare utilization and costs were mainly driven by DMTs, whereas we did not account for symptomatic treatments. However, symptomatic treatments are an unfortunate consequence of MS progression and, as such, improved management of DMTs could also impact positively on symptomatic treatments. Additional medical costs (e.g., initial assessment and differential diagnosis) are negligible when compared with DMT prescription and management [28]. Not least, indirect costs should have been evaluated in order to have a full view on MS-related costs [4]. In the future, a more comprehensive approach should be considered, with the inclusion not only of patients regularly followed-up at the MS centre for DMT management, but also of patients with higher disability only seen by local services. We decided to exclude patients who took part in clinical trials where expenses for DMT prescription and management are generally covered by the funder, with risk of cost underestimation. Data were acquired in a limited but relatively homogenous geographical and healthcare area, as we aimed to obtain a comprehensive view on healthcare utilization, and their generalizability to other populations would deserve further investigations (of note, selected MS Centres included 80% MS patients within ASL NA3 SUD). Our heterogenous MS cohort presented with variable disease course, activity and disability, that were accounted for in our statistical models. In particular, we adjusted our statistical models for baseline EDSS and follow-up duration, that were different between two Centres and, as such, could imply less relapses and lower costs. Finally, we did not perform any sub-analysis for different DMTs due to sample size constraints; but, differences between DMTs would be determined mainly by different DMT costs, than healthcare organization [2,3].

In conclusion, the MS Care Unit is the ideal model of multidisciplinary care in MS from neurologists’ point of view [8], but its economic viability remains to be further explored in the long term and in relation to patient preference. A balanced and coordinated integration of local and centralised services should be considered during the formation of MS Care Units. Not least, a specific care should be given to patient more at risk of relapses and disability progression in order to prevent higher costs (and worse disability).
